# Primary care telehealth utilization by access-challenged populations in Medicare Advantage

**DOI:** 10.1093/haschl/qxae120

**Published:** 2024-09-28

**Authors:** Emily Boudreau, Amanda Sutherland, Debra Bozzi, Melanie Canterberry, Gosia Sylwestrzak

**Affiliations:** Humana Healthcare Research, Louisville, KY 40202, United States; Humana Healthcare Research, Louisville, KY 40202, United States; Humana Healthcare Research, Louisville, KY 40202, United States; Humana Healthcare Research, Louisville, KY 40202, United States; Humana Healthcare Research, Louisville, KY 40202, United States

**Keywords:** telehealth, primary care, access, value-based payment

## Abstract

Although telehealth utilization in primary care has decreased markedly since 2020, it remains higher than before the COVID-19 pandemic. There is debate about its role in a post-pandemic healthcare system, particularly for certain groups of patients that may experience greater access challenges related to in-person care. To inform this debate, we examined the use of audiovisual telehealth for primary care as a share of total primary care outpatient visits among Medicare Advantage beneficiaries with and without 3 characteristics associated with potential access challenges (low-income status, disability, and frailty). Primary care visits when the beneficiary was frail were 39.4% (OR: 1.39 [95% CI, 1.37-1.42]) more likely to be telehealth; when the beneficiary was disabled or low-income status, visits were 20.1% (OR: 1.20 [95% CI, 1.18-1.22]) and 8.3% (OR: 1.08 [95% CI, 1.05-1.12]) more likely to be telehealth, respectively. The differential use of telehealth among beneficiaries with low-income status or disability, compared with those without, was significantly larger among providers with a 2-sided risk contract compared with fee for service (low-income status: OR: 1.19 [95% CI, 1.04-1.35]; disability: OR: 1.07 [95% CI, 1.01-1.13]).

## Introduction

During the COVID-19 pandemic, telehealth utilization in primary care grew to unprecedented levels.^1^ Although its use remains higher than before the pandemic, it has decreased markedly since 2020. Debate remains about its role in a post-pandemic healthcare system, particularly for certain groups of patients that may experience greater access challenges related to in-person care, where the evidence on its use and impact has been conflicting.^2-4^

Within the Medicare program, the Consolidated Appropriations Act of 2023 extended pandemic-era telehealth flexibilities through December 31, 2024. A more thorough understanding of telehealth utilization in Medicare Advantage (MA) would help inform evidenced-based telehealth policy beyond 2024.

Using Humana claims data from 2021 to 2023, we examined the use of audiovisual telehealth for primary care outpatient care among MA beneficiaries with and without 3 characteristics associated with potential access challenges (low-income status, disability, and frailty). With increasing evidence that provider characteristics influence patient access to telehealth,^5-8^ we also assessed whether primary care providers engaged in value-based arrangements in MA utilized telehealth differently than other providers for these access-challenged groups of beneficiaries. Under value-based care, reimbursement is tied to specific cost and quality measures, thereby incentivizing providers to offer high-quality care at minimal cost. This framework may encourage providers to offer care modalities to facilitate primary care visits for patients with different needs and preferences—including telehealth—to manage and prevent chronic conditions.

## Study data and methods

### Data and study population

Using Humana claims data from January 2021 to March 2023, we identified monthly cohorts of MA beneficiaries enrolled in Health Maintenance Organization (HMO) and Preferred Provider Organization (PPO) plans. Eligible beneficiaries were included for each month of the study period they were enrolled in a plan. Beneficiaries were excluded from the study if their plan was contractually excluded from research or their assigned primary care provider delegated claims to a third party, since the MA plan administrator does not have full access to delegated claims. Beneficiaries were excluded if they were in institutionalized care during the study period and for months after entering hospice, as the MA plan administrator does not have access to claims during these months. Additional detail on the cohort is available in a patient waterfall diagram in the Supplementary material.

### Potential access challenges

We identified beneficiaries with and without 3 potential access challenges to primary care: low-income status, disability, or frailty. Low-income status was determined monthly and defined as Medicare–Medicaid dual-eligible status or receipt of Medicare Part D low-income subsidy. Disability was defined using the original reason for Medicare entitlement. Frailty was assessed using a validated medical claims-based frailty index, which relies on 12 months of prior enrollment.^9^ Therefore, beneficiaries with enrollment periods shorter than 12 months were not included in frailty analyses. The frailty index provides a continuous score from 0 to 1. We categorized frail as an index score ≥ 0.25 and nonfrail as an index score < 0.25. The 3 subpopulations were not mutually exclusive, and beneficiaries could belong to more than 1 subpopulation in a month. Additional detail is available in the Supplementary material on the overlap between access-challenged subpopulations.

### Primary care organization payment arrangement

We identified the primary care provider organization to which each beneficiary was assigned through health plan data. Beneficiaries either select a primary care provider organization or they are attributed to one by the health plan based on visit and other claims data over the previous 24 months. Additional detail on beneficiary assignment to primary care provider organization is available in the Supplementary material.

Then, we used contract data to identify the payment model under which the primary care provider was reimbursed by the MA plan for the beneficiaries’ care and classified those payment models according to the following taxonomy: fee for service (FFS); shared savings with upside-only financial risk (upside-only risk); and shared savings with upside and downside financial risk (2-sided risk). Shared savings models with upside financial risk give providers the opportunity to earn a bonus payment for meeting certain quality and spending criteria. Those with both upside and downside risks can earn similar bonus payments or owe a financial deficit to the health plan for failing to adequately manage the patient population for which they are responsible. The contract data reflect the payment arrangement that the provider organization was in during the month that the assigned beneficiary was in the analysis.

### Covariates

Beneficiary-level covariates included age, sex, race, and rural status. Race was assessed according to the Centers for Medicare and Medicaid Services (CMS) beneficiary race code and categorized as White, Black, other (Asian, Hispanic, North American Native, and Other), and unknown. Rural status was defined using a Rural–Urban Commuting Area ≥ 4 based on residential zip code.

### Telehealth use

We first used claims to identify in-person and audiovisual telehealth outpatient encounters with a primary care provider using a combination of place of service codes, procedure codes for clinician visits, and provider specialty. Among the identified outpatient, primary care encounters we classified visits as audiovisual telehealth using place of service codes 02 or 10 and procedure modifier codes of 95, GT, or GQ. Additional detail on the outpatient and telehealth visit identification logic is included in the Supplementary material. Audiovisual telehealth visits were selected for this analysis for 3 main reasons: (1) audio-only visits have been investigated more thoroughly in similar, marginalized populations previously^10-12^; (2) greater uncertainty about how potential policy shifts at the end of 2024 will affect the availability and use of audio-only telehealth, as some pandemic-era telehealth flexibilities are set to expire; and (3) concerns about the clinical quality of audio-only visits.^13^

### Analytic approach

We first descriptively examined the use of telehealth for primary care as a share of total primary care outpatient visits each month and visually compared trends in telehealth utilization over time among those with and without the 3 access challenges.

Next, we constructed 2 visit-level generalized linear regression models to examine the association between having low-income status, disability, or frailty and telehealth utilization. We used a binomial distribution with a logit link function. The outcome for each model was whether the primary care visit was telehealth or not. Each model included all 3 patient access barriers and controlled for patient age, sex, race, state, and rural status. Month fixed effects were included in both models, and standard errors were clustered by the primary care provider Tax Id offering the visit. The models differed in the cohort evaluated; because the frailty index we used to assess frailty required 12 months of continuous enrollment, the model focused on the effect of frailty was based on a smaller cohort.

To assess the role of primary care provider participation in upside-only and 2-sided risk contracts (compared with FFS), 3 separate models were constructed, one for each access challenge (low-income status, disability, and frailty). These models included an interaction between the access challenge and the primary care provider payment arrangement. We controlled for the same variables as the earlier models and included month fixed effects with standard errors clustered by the primary care provider Tax Id offering the visit.

All regression equations are included in the Supplementary material.

## Results

After study exclusions, the final study population included 3 859 626 MA beneficiaries during the 2021-2023 time period. 32.2% were low-income status, 35.2% were disabled, and 9.0% were frail at some point over the study period. 35.8% of beneficiaries were attributed to a primary care provider in a FFS arrangement, 44.5% in an upside-only risk arrangement, and 19.7% in a 2-sided risk arrangement (Table 1).

**Table 1.** Beneficiary characteristics.

	Full cohort	Low-income status	Without low-income status	SMD	Disability	Without disability	SMD	Frailty	Without frailty	SMD
Sample size, *N* (%)	3 859 626 (100.0)	1 242 067 (32.2)	2 617 559 (67.8)		1 358 423 (35.2)	2 501 203 (64.8)		348 100 (12.1)	2 518 636 (87.9)	
Age; mean (SD)^c^	71 (10.3)	66 (11.9)	73 (8.5)	−0.67	63 (10.5)	75 (7.2)	−1.35	73 (11.0)	72 (10.0)	0.09
Female, *N* (%)	2 200 703 (57.0)	783 455 (63.1)	1 417 248 (54.1)	0.18	753 272 (55.5)	1 447 431 (57.9)	−0.05	230 369 (66.2)	1 403 403 (55.7)	0.21
Race, *N* (%)										
White	2 836 505 (73.5)	764 675 (61.6)	2 071 830 (79.2)	−0.38	941 000 (69.3)	1 895 505 (75.8)	−0.15	273 623 (78.6)	1 900 800 (75.5)	0.07
Black	740 458 (19.2)	378 697 (30.5)	361 761 (13.8)	0.4	339 851 (25.0)	400 607 (16.0)	0.22	62 021 (17.8)	451 457 (17.9)	0
Other	188 803 (4.9)	79 909 (6.4)	108 894 (4.2)	0.1	56 300 (4.1)	132 503 (5.3)	−0.05	9865 (2.8)	116 681 (4.6)	−0.09
Unknown	93 860 (2.4)	18 786 (1.5)	75 074 (2.9)	−0.09	21 272 (1.6)	72 588 (2.9)	−0.09	2591 (0.7)	49 698 (2.0)	−0.11
Rural, *N* (%)^a^	1 048 798 (27.2)	390 069 (31.4)	658 729 (25.2)	0.14	430 734 (31.7)	618 064 (24.7)	0.16	97 816 (28.1)	668 965 (26.6)	0.03
Low-income status, *N* (%)^a^	1 242 067 (32.2)	n/a	n/a		732 062 (53.9)	510 005 (20.4)	0.66	156 740 (45.0)	689 697 (27.4)	0.36
Disability, *N* (%)^a^	1 358 423 (35.2)	732 062 (58.9)	626 361 (23.9)	0.67	n/a	n/a		170 120 (48.9)	812 040 (32.2)	0.33
Frailty, *N* (%)^a^	348 100 (9.0)	156 740 (12.6)	191 360 (7.3)	0.18	170 120 (12.5)	177 980 (7.1)	0.18	n/a	n/a	
Primary care provider payment arrangement, *N* (%)^b^										
Fee for service	1 383 246 (35.8)	481 653 (38.8)	901 593 (34.4)	0.09	518 760 (38.2)	864 486 (34.6)	0.08	103 858 (29.8)	815 953 (32.4)	−0.06
Upside-only risk	1 716 727 (44.5)	532 343 (42.9)	1 184 384 (45.3)	−0.05	595 277 (43.8)	1 121 450 (44.8)	−0.02	165 018 (47.4)	1 178 500 (46.8)	0.01
Two-sided risk	759 653 (19.7)	228 071 (18.4)	531 582 (20.3)	−0.05	244 386 (18.0)	515 267 (20.6)	−0.07	79 224 (22.8)	524 183 (20.8)	0.05
Plan type, *N* (%)^b^										
HMO^d^	1 866 987 (48.4)	685 716 (55.2)	1 181 271 (45.1)	0.20	672 635 (49.5)	1 194 352 (47.8)	0.04	183 398 (52.7)	1 240 130 (49.2)	0.07
PPO^e^	1 992 639 (51.6)	556 351 (44.8)	1 436 288 (54.9)	−0.20	685 788 (50.5)	1 306 851 (52.3)	−0.04	164 702 (47.3)	1 278 506 (50.8)	−0.07

Table 1 is presented at the unique beneficiary level. For characteristics that could change over the study time period, see below.

^a^If beneficiary met the criteria at least once, then reflected in Table 1.

^b^ Table 1 reflects the majority of time.

^c^ Table 1 reflects the maximum value.

^d^Health Maintenance Organization.

^e^Preferred Provider Organization.

**Figure 1.**
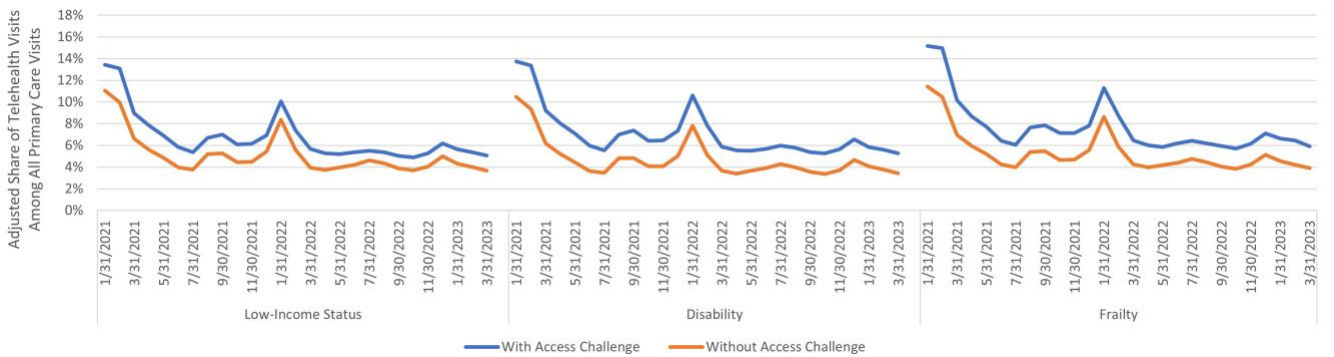
Trends over time by potential access challenge.

Descriptively, beneficiaries with low income, disability, or frailty consistently used telehealth as a greater share of primary care visits than those without those access challenges across the study time period. Patterns of telehealth utilization over the study time period were similar for beneficiary groups with and without potential access challenges across all 3 subpopulations (Figure 1) and were consistent with seasonal differences and surges in COVID-19 across the time period (eg, the Omicron COVID-19 variant in early 2022).

**Figure 2.**
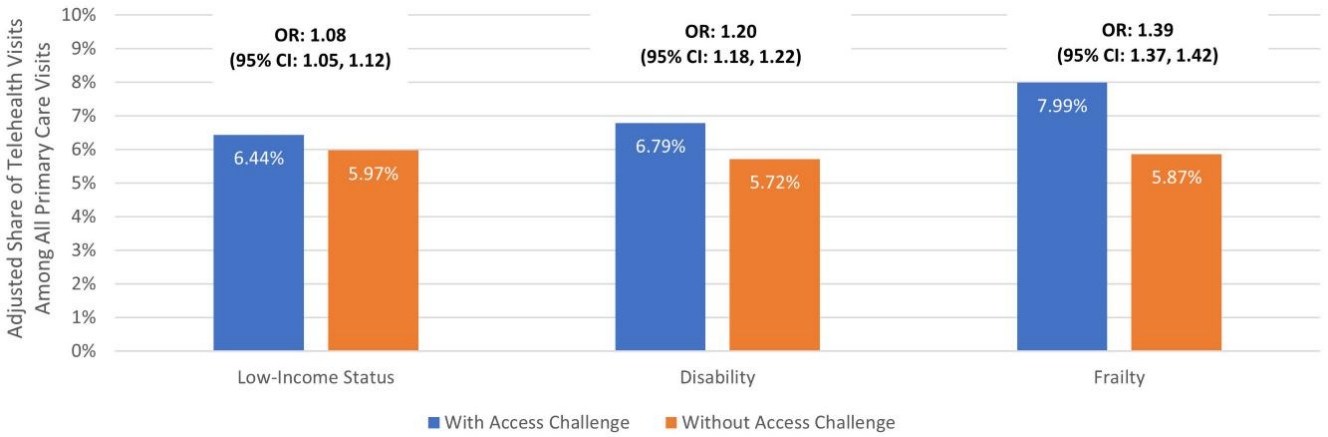
Adjusted telehealth utilization by groups with and without potential access challenges as a share of all primary care encounters from January 2021 to March 2023.

In adjusted models, telehealth visits occurred more frequently among all 3 potentially access-challenged groups relative to those without (Figure 2). Primary care visits when the beneficiary was frail were 39.4% (OR: 1.39 [95% CI, 1.37-1.42]) more likely to be telehealth; when the beneficiary was disabled or low-income status, visits were 20.1% (OR: 1.20 [95% CI, 1.18-1.22]) and 8.3% (OR: 1.08 [95% CI, 1.05-1.12]) more likely to be telehealth, respectively.

**Figure 3.**
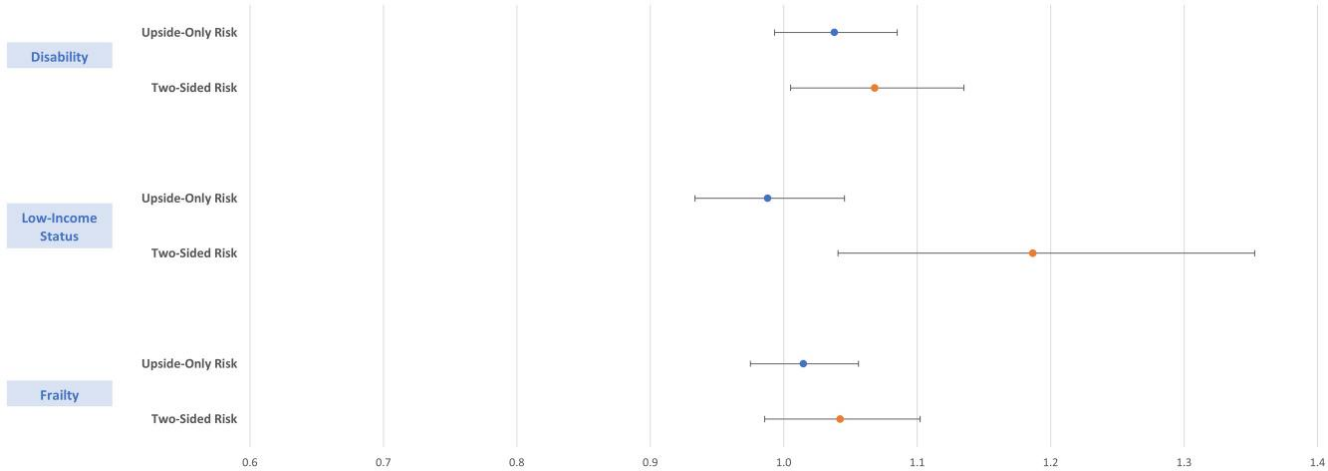
Odds of telehealth utilization by provider payment arrangement (compared with FFS) for potentially access-challenged beneficiaries.

Each model was repeated with an interaction between the access challenge and primary care provider payment arrangement. The differential use of telehealth among beneficiaries with low-income status or disability, compared with those without, was significantly larger among providers with a 2-sided risk contract compared with FFS (low-income status: OR: 1.19 [95% CI, 1.04-1.35]; disability: OR: 1.07 [95% CI, 1.01-1.13]) **(**Figure 3). There was no statistically significant differential use of telehealth among beneficiaries with frailty, disability, or low-income status for upside-only providers compared with FFS.

## Discussion

In this evaluation of nearly 4 million MA beneficiaries, we examined whether beneficiaries with potential healthcare access challenges use telehealth differently and found that primary care visits when the beneficiary was frail, disabled, or low income were 39.4%, 20.1%, and 8.3% more likely to be telehealth, respectively, compared with individuals without those characteristics. Moreover, the differential use of telehealth among beneficiaries with low-income status and disability was 18.7% and 7.0% greater, respectively, among providers with a 2-sided risk contract compared with FFS.

Although originally telehealth was primarily covered as a means to improve access to care for patients living in rural areas, expansion in payment policy has provided the opportunity to more broadly benefit patient groups who have traditionally faced challenges accessing care. Particularly with the recently expanded access to audiovisual telehealth as a result of the COVID-19 pandemic, we have limited understanding of how individuals with traditional barriers to care—who may be likely to benefit from the enhanced access telehealth provides—utilize telehealth. Our findings that beneficiaries with 3 such characteristics—low-income status, disability, and frailty—were more likely to use audiovisual telehealth for primary care than those without suggest that telehealth meets a need for those beneficiaries. For instance, frail or disabled beneficiaries may have mobility issues that make it difficult to leave their homes, while low-income beneficiaries may be more likely to face transportation challenges related to cost. Primary care providers may differ in their use of care management strategies, including the differential use of telehealth, to support these access-challenged populations.

These findings are consistent with a recent CMS brief, which reported that traditional Medicare beneficiaries with disability or dual Medicare–Medicaid eligibility (ie, low-income status) used more telehealth than those without these potential access challenges.^14^ The present study furthers and strengthens those findings, as CMS's descriptive analysis did not evaluate frailty or adjust for other beneficiary characteristics.

This study focused on the use of audiovisual visits (ie, those with video capability) as opposed to audio-only visits (ie, those with telephone only and no video capability). Previous research has found that access-challenged or marginalized populations may be more likely to use audio-only telehealth compared with audiovisual telehealth.^10-12^ However, none of these analyses have focused in-depth on how these populations use audiovisual telehealth. This is particularly important to understand for 2 reasons. First, concerns have been raised about the potential for lower clinical quality provided by audio-only visits.^13^ Second, audiovisual telehealth use may more accurately represent future telehealth use, regardless of potential Medicare reimbursement changes. Medicare coverage of audio-only telehealth visits was first adopted as part of the COVID-19 public health emergency in 2020; however, most of the coverage expansions are temporary and set to expire at the end of 2024, including those related to audio-only telehealth visits for most primary care. Conversely, reimbursement for audiovisual telehealth visits is expected to continue beyond 2024, although rates and types of visit reimbursements remain uncertain.

Our results are consistent with other recent analyses that suggest provider characteristics, including participation in a value-based risk arrangement, influence telehealth usage.^5-8^ A 2021 study on the use of telehealth during the height of the COVID-19 pandemic found that among primary care providers engaged in risk contracts in MA, telehealth use rose faster and reached higher absolute levels than in FFS contracts.^6^ The current analysis extends that study's findings into a time period when COVID-19 vaccines were available, other services and social gatherings had opened, and patient and provider behavior is more likely to have returned to normal.

Furthermore, this study adds to a growing body of literature that demonstrates that providers are more sensitive to losses than gains in value-based risk arrangements.^15,16^ The current analysis finds that providers in 2-sided risk arrangements (ie, those encompassing both financial gains and penalties) are more likely to provide telehealth to beneficiaries with low-income status or disability, compared with those without. The same result is not observed for providers in upside-only risk contracts, suggesting that financial penalties may encourage effective care management, as they incentivize higher use of telehealth primary care, especially among beneficiaries with access challenges.

Most notably, this study extends our understanding of telehealth use by suggesting that a combination of beneficiary and provider factors influences the use of telehealth. Our analysis indicates that telehealth could be meeting a need among patients with barriers to traditional healthcare who may require special consideration in future policy changes. Additionally, certain providers may be better able to accommodate or encourage telehealth as an option for primary care for these patients. Results suggest that primary care providers engaged in 2-sided risk may be using telehealth as a care management tool for the most vulnerable patients.

This analysis had several limitations. First, this study was limited to MA beneficiaries in 1 national health plan. Although the health plan serves nearly a fifth of the MA population, the findings may not reflect trends observed in other demographic cohorts, including traditional Medicare. Second, we did not include a measure of clinical risk in the adjusted models as doing so would have necessitated a continuous enrollment requirement to construct a baseline period, thus reducing the study population. Third, this study was observational in design and any potential causal relationships cannot be determined. Finally, we were limited in the primary care provider characteristics we could control for due to data limitations.

Future analyses should continue to explore the range of provider characteristics that might influence the use of telehealth (eg, patient visit volume, provider group size, or affiliation with larger systems). Additionally, research should continue to emphasize whether outcomes from primary care telehealth encounters are equivalent to in-person care, particularly given that certain, potentially vulnerable, groups of patients may rely more heavily on them.

## Supplementary Material

qxae120_Supplementary_Data

## Data Availability

The authors do not have permission to share the data.
