# Supplementary material for: Primary care telehealth utilization by access-challenged populations in Medicare Advantage
Source: Health Aff Sch. 2024 Sep 28;2(10):qxae120. doi: 10.1093/haschl/qxae120 (PMC11465365; doi:10.1093/haschl/qxae120)
Supplement: qxae120_Supplementary_Data [file qxae120_supplementary_data.zip › Supplement.docx]

**Supplemental Material**

**Section 1 Additional Detail on Beneficiary Cohort**

1a. Patient Waterfall

2a. State Breakdown

3a. Overlap Among Access-Challenged Subpopulations

**Section 2 Beneficiary / Primary Care Provider Group Assignment and Value-Based Care Contract Attribution**

**Section 3 Visit Identification Strategy**

3a. Primary Care Visit Identification

3b. Telehealth Visit Identification (Subset of Primary Care Visits)

**Section 4 Regression Equations**

**Section 1** **Additional Detail on Beneficiary Cohort**

*Patient Waterfall Diagram*

Humana MA Beneficiaries between January 2021 and March 2023

7,596,727

**Excluded from analysis***

Beneficiaries Contractually Excluded from Research (1,126,541)

Beneficiaries with Primary Care Providers that Delegate Claims (515,203)

Beneficiaries without Contractual Research Exclusions or Providers that Delegate Claims

5,994,654

Beneficiaries without institutional care or hospice for the full study period**

5,937,267

**Main Analytical Cohort**

Beneficiaries with at least one outpatient primary care visit during the study period

3,859,626

* *Beneficiaries could qualify for both exclusions (delegated claims and contractual research exclusions)*

**For Frail vs. Non-Frail analysis only*****

Beneficiaries with at least 1-year of Continuous Enrollment

2,866,736

***Beneficiaries with hospice for only part of the study period were included for those months where they were not in institutional care or hospice; this line excludes only those beneficiaries who were in hospice for the full duration of the study period*

****The frailty index we used required one year of continuous enrollment prior to the study period to identify the frail and non-frail beneficiaries*

**State Representation**

| State | Beneficiaries (N) | Percent of Study Population (%) |
| --- | --- | --- |
| TX | 333672 | 8.65 |
| GA | 291117 | 7.54 |
| FL | 258126 | 6.69 |
| NC | 241868 | 6.27 |
| TN | 172930 | 4.48 |
| OH | 169051 | 4.38 |
| LA | 168446 | 4.36 |
| VA | 162712 | 4.22 |
| SC | 162583 | 4.21 |
| IL | 149759 | 3.88 |
| IN | 144925 | 3.75 |
| KY | 139856 | 3.62 |
| AZ | 120405 | 3.12 |
| MI | 116385 | 3.02 |
| MS | 109755 | 2.84 |
| NY | 109255 | 2.83 |
| AL | 102844 | 2.66 |
| PA | 87936 | 2.28 |
| MO | 78346 | 2.03 |
| OK | 73656 | 1.91 |
| CO | 71143 | 1.84 |
| AR | 69636 | 1.8 |
| MN | 64910 | 1.68 |
| WI | 58239 | 1.51 |
| WV | 52562 | 1.36 |
| WA | 52182 | 1.35 |
| NM | 37257 | 0.97 |
| KS | 35458 | 0.92 |
| MT | 33185 | 0.86 |
| IA | 28546 | 0.74 |
| NJ | 23768 | 0.62 |
| NV | 19774 | 0.51 |
| UT | 19366 | 0.5 |
| OR | 15956 | 0.41 |
| DE | 13617 | 0.35 |
| NH | 10666 | 0.28 |
| ID | 10561 | 0.27 |
| ME | 9917 | 0.26 |
| MD | 9901 | 0.26 |
| NE | 8108 | 0.21 |
| SD | 8019 | 0.21 |
| ND | 3804 | 0.1 |
| MA | 3563 | 0.09 |
| CA | 2833 | 0.07 |
| CT | 1776 | 0.05 |
| WY | 427 | 0.01 |
| VT | 377 | 0.01 |
| RI | 153 | 0 |
| AK | 116 | 0 |
| HI | 113 | 0 |
| DC | 66 | 0 |

**Overlap among access-challenged subpopulations**

| Cohort | Beneficiaries (N) |
| --- | --- |
| Low-Income Status | 1,242,067 |
| Disability | 1,358,423 |
| Frail | 348,100 |
| Low-Income Status & Disability | 732,062 |
| Low-Income Status & Frail | 156,740 |
| Disability & Frail | 170,120 |
| Low-Income Status, Disability, and Frail | 101,374 |

*Note: Beneficiary counts reflect beneficiaries who fell into that access-challenged population for at least one month during the study period.*

**Section 2 Beneficiary / Primary Care Provider Group Assignment and Value-Based Care Contract Attribution**

Every Humana MA beneficiary receives an assigned primary care provider organization (Tax ID level assignment). For HMO plans, beneficiaries are required to select a primary care provider organization. For PPO plans, beneficiaries may also select a primary care provider organization, but are not required to do so. If the beneficiary does not select a primary care provider organization, attribution to one occurs based on claims for evaluation/management (E/M) visits, wellness visits, physical assessments and some OB-GYN visits. Visits are evaluated within the most recent six months, then the remainder of a year, then 13-24 months. In the event of a tie, total visits, most recent visit date and total claim dollars are used. Beneficiaries must have three or more consecutive visits to cause a change in the attributed primary care provider organization. Patients who currently do not have an assigned primary care provider organization will be attributed with one visit. Patients can call Humana at any point to change their assigned primary care provider organization.

Then, we used contract data to identify the payment model under which the primary care provider was reimbursed by the MA plan for the beneficiary’s care and classified those payment models according to the following taxonomy: fee-for-service (FFS); shared savings with upside-only financial risk (upside-only risk); and shared savings with upside and downside financial risk (two-sided risk). Shared savings models with upside financial risk give providers the opportunity to earn a bonus payment for meeting certain quality and spending criteria. Those with both upside and downside risk can earn similar bonus payments or owe a financial deficit to the health plan for failing to appropriately manage the patient population for which they are responsible. The contract data reflects the payment arrangement that the provider organization was in for a given beneficiary during the month in the analysis.

**Section 3 In-Person and Telehealth Primary Care Visit Identification Strategy**

*Primary Care Visit Identification*

This analysis used outpatient claims for a clinician visit with a primary care provider that were identified as either in-person or telehealth.*

1. Outpatient claims were identified by POS codes (listed below).
2. Clinician visits were identified by CPT/HCPCS codes (listed below).
3. Clinician visits delivered by a primary care practitioner were determined by the billing NPI’s HIPPA provider taxonomy (included classification and specializations listed below).
4. Audio-visual telehealth visits were determined by POS or CPT modifier (listed below).

*All claims were considered for primary care visit identification, regardless of the beneficiary’s assigned provider-group at the time.

| **Outpatient Claims** | **Clinician Visits** |
| --- | --- |
| **POS Codes** | **CPT/HCPCS** |
| **02** | **G0101** |
| **05** | **G0245** |
| **06** | **G0248** |
| **10** | **G0402** |
| **11** | **0500F** |
| **12** | **99201** |
| **13** | **99202** |
| **14** | **99203** |
| **20** | **99204** |
| **22** | **99205** |
| **24** | **99381** |
| **32** | **99382** |
| **33** | **99383** |
| **34** | **99384** |
| **49** | **99385** |
| **50** | **99386** |
| **71** | **99387** |
|  | **G0246** |
|  | **G0247** |
|  | **G0250** |
|  | **G0420** |
|  | **G0421** |
|  | **G0463** |
|  | **0502F** |
|  | **0503F** |
|  | **1000F** |
|  | **2000F** |
|  | **95115** |
|  | **95117** |
|  | **99058** |
|  | **99211** |
|  | **99212** |
|  | **99213** |
|  | **99214** |
|  | **99215** |
|  | **99354** |
|  | **99355** |
|  | **99366** |
|  | **99367** |
|  | **99391** |
|  | **99392** |
|  | **99393** |
|  | **99394** |
|  | **99395** |
|  | **99396** |
|  | **99397** |
|  | **99401** |
|  | **99402** |
|  | **99403** |
|  | **99404** |
|  | **99411** |
|  | **99412** |
|  | **99420** |
|  | **99429** |

| **Primary Care** | |
| --- | --- |
| **HIPPA Taxonomy Class/Specialization Combinations** | |
| **Class** | **Specialization** |
| **General Practice** |  |
| **Nurse Practitioner** |  |
| **Physician Assistant** |  |
| **Family Medicine** |  |
| **Internal Medicine** | **No Specialization (must be blank)** |
| **Internal Medicine** | **Geriatric Medicine** |
|  | **Community Health** |
|  | **Primary Care** |
|  | **Public Health, Federal** |
|  | **Public Health, State of Local** |

***Audio-Visual Telehealth Visit Identification (Subset of Primary Care Visits)***

| **Telehealth Visits** | |
| --- | --- |
| **POS** | **CPT Modifier** |
| **2** | **95** |
| **10** | **GT** |
|  | **GQ** |

Note: Audio-only visits (CPT codes 99441-99443) were not included.

**Section 4 Regression Equations**

***Variables:***

- **Telehealth Use** $\boldsymbol{(}\boldsymbol{Y}_{\boldsymbol{it}}\boldsymbol{)}$ **=** Binary outcome variable where 1 represents a telehealth visit and 0 represents a non-telehealth visit
- **Disability =** Binary indicator where 1 represents disability as the original reason for Medicare entitlement
- **Age =** Continuous variable for beneficiary age at the start of a given calendar year
- **Gender =** Categorical variable for beneficiary gender
- **Race =** Categorical variable for beneficiary race
- **Rural =** Binary indicator where 1 represents beneficiaries living in rural areas in a given month
- **Frail =** Categorical variable for beneficiary frailty in a given year
- **Low-Income Status (LIS/DE)=** Binary indicatory where 1 represents beneficiaries eligible to receive a low-income subsidy and/or dually eligible for Medicare and Medicaid in a given month
- **VBC:** Categorized as FFS, One-Sided Risk, or Two-Sided Risk based on payment arrangement for that beneficiary for their assigned provider group in a given month
- **State:** Categorical variable for beneficiary state

**Indices:**

- i = Beneficiary-level
- t = Month-level

**Notes (all models)**

- standards errors clustered by visit Tax ID
- time fixed effects (month)

| **Disability**  **Low-Income Status** | $\boldsymbol{Y}_{\boldsymbol{it}}\boldsymbol{=}\boldsymbol{B}_{\boldsymbol{0}}\boldsymbol{+}\boldsymbol{B}_{\boldsymbol{1}}\boldsymbol{\cdot}\boldsymbol{Disability}_{\boldsymbol{i}}\boldsymbol{+}\boldsymbol{B}_{\boldsymbol{2}}\boldsymbol{\cdot}\boldsymbol{Age}_{\boldsymbol{it}}\boldsymbol{+}\boldsymbol{B}_{\boldsymbol{3}}\boldsymbol{\cdot}\boldsymbol{Gender}_{\boldsymbol{i}}\boldsymbol{+}\boldsymbol{B}_{\boldsymbol{4}}\boldsymbol{\cdot}\boldsymbol{Race}_{\boldsymbol{i}}\boldsymbol{+}\boldsymbol{B}_{\boldsymbol{5}}\boldsymbol{\cdot}\boldsymbol{Rural}_{\boldsymbol{it}}\boldsymbol{+}\boldsymbol{B}_{\boldsymbol{6}}\boldsymbol{\cdot}\boldsymbol{Frail}_{\boldsymbol{it}}\boldsymbol{+}\boldsymbol{B}_{\boldsymbol{7}}\boldsymbol{\cdot}\boldsymbol{LIS/DE}_{\boldsymbol{it}}\boldsymbol{+}\boldsymbol{B}_{\boldsymbol{8}}\boldsymbol{\cdot}\boldsymbol{Month}_{\boldsymbol{t}}\boldsymbol{+}\boldsymbol{B}_{\boldsymbol{1}}\boldsymbol{\cdot}\boldsymbol{State}_{\boldsymbol{it}}$ |
| --- | --- |
| **Frail *** | $\boldsymbol{Y}_{\boldsymbol{it}}\boldsymbol{=}\boldsymbol{B}_{\boldsymbol{0}}\boldsymbol{+}\boldsymbol{B}_{\boldsymbol{1}}\boldsymbol{\cdot}\boldsymbol{Frail}_{\boldsymbol{it}}\boldsymbol{+}\boldsymbol{B}_{\boldsymbol{2}}\boldsymbol{\cdot}\boldsymbol{Age}_{\boldsymbol{it}}\boldsymbol{+}\boldsymbol{B}_{\boldsymbol{3}}\boldsymbol{\cdot}\boldsymbol{Gender}_{\boldsymbol{i}}\boldsymbol{+}\boldsymbol{B}_{\boldsymbol{4}}\boldsymbol{\cdot}\boldsymbol{Race}_{\boldsymbol{i}}\boldsymbol{+}\boldsymbol{B}_{\boldsymbol{5}}\boldsymbol{\cdot}\boldsymbol{Rural}_{\boldsymbol{it}}\boldsymbol{+}\boldsymbol{B}_{\boldsymbol{6}}\boldsymbol{\cdot}\boldsymbol{Disability}_{\boldsymbol{i}}\boldsymbol{+}\boldsymbol{B}_{\boldsymbol{7}}\boldsymbol{\cdot}\boldsymbol{LIS/DE}_{\boldsymbol{it}}\boldsymbol{+}\boldsymbol{B}_{\boldsymbol{8}}\boldsymbol{\cdot}\boldsymbol{Month}_{\boldsymbol{t}}\boldsymbol{+}\boldsymbol{B}_{\boldsymbol{1}}\boldsymbol{\cdot}\boldsymbol{State}_{\boldsymbol{it}}$ |

**Note: Cohort differed due to continuous enrollment requirement for frailty cohort.*

**Models with Interactions Between Access Challenge and Payment Arrangement**

| **Disability x VBC** | $\boldsymbol{Y}_{\boldsymbol{it}}\boldsymbol{=}\boldsymbol{B}_{\boldsymbol{0}}\boldsymbol{+}\boldsymbol{B}_{\boldsymbol{1}}\boldsymbol{\cdot}\boldsymbol{Disability}_{\boldsymbol{i}}\boldsymbol{\cdot}\boldsymbol{VBC}_{\boldsymbol{it}}\boldsymbol{+}\boldsymbol{B}_{\boldsymbol{2}}\boldsymbol{\cdot}\boldsymbol{Age}_{\boldsymbol{it}}\boldsymbol{+}\boldsymbol{B}_{\boldsymbol{3}}\boldsymbol{\cdot}\boldsymbol{Gender}_{\boldsymbol{i}}\boldsymbol{+}\boldsymbol{B}_{\boldsymbol{4}}\boldsymbol{\cdot}\boldsymbol{Race}_{\boldsymbol{i}}\boldsymbol{+}\boldsymbol{B}_{\boldsymbol{5}}\boldsymbol{\cdot}\boldsymbol{Rural}_{\boldsymbol{it}}\boldsymbol{+}\boldsymbol{B}_{\boldsymbol{6}}\boldsymbol{\cdot}\boldsymbol{Frail}_{\boldsymbol{it}}\boldsymbol{+}\boldsymbol{B}_{\boldsymbol{7}}\boldsymbol{\cdot}\boldsymbol{LIS/DE}_{\boldsymbol{it}}\boldsymbol{+}\boldsymbol{B}_{\boldsymbol{8}}\boldsymbol{\cdot}\boldsymbol{Month}_{\boldsymbol{t}}\boldsymbol{+}\boldsymbol{B}_{\boldsymbol{1}}\boldsymbol{\cdot}\boldsymbol{State}_{\boldsymbol{it}}$ |
| --- | --- |
| **LIS/DE x VBC** | $\boldsymbol{Y}_{\boldsymbol{it}}\boldsymbol{=}\boldsymbol{B}_{\boldsymbol{0}}\boldsymbol{+}\boldsymbol{B}_{\boldsymbol{1}}\boldsymbol{\cdot}\boldsymbol{LIS/DE}_{\boldsymbol{it}}\boldsymbol{\cdot}\boldsymbol{VBC}_{\boldsymbol{it}}\boldsymbol{+}\boldsymbol{B}_{\boldsymbol{2}}\boldsymbol{\cdot}\boldsymbol{Age}_{\boldsymbol{it}}\boldsymbol{+}\boldsymbol{B}_{\boldsymbol{3}}\boldsymbol{\cdot}\boldsymbol{Gender}_{\boldsymbol{i}}\boldsymbol{+}\boldsymbol{B}_{\boldsymbol{4}}\boldsymbol{\cdot}\boldsymbol{Race}_{\boldsymbol{i}}\boldsymbol{+}\boldsymbol{B}_{\boldsymbol{5}}\boldsymbol{\cdot}\boldsymbol{Rural}_{\boldsymbol{it}}\boldsymbol{+}\boldsymbol{B}_{\boldsymbol{6}}\boldsymbol{\cdot}\boldsymbol{Frail}_{\boldsymbol{it}}\boldsymbol{+}\boldsymbol{B}_{\boldsymbol{7}}\boldsymbol{\cdot}\boldsymbol{Disability}_{\boldsymbol{i}}\boldsymbol{+}\boldsymbol{B}_{\boldsymbol{8}}\boldsymbol{\cdot}\boldsymbol{Month}_{\boldsymbol{t}}\boldsymbol{+}\boldsymbol{B}_{\boldsymbol{1}}\boldsymbol{\cdot}\boldsymbol{State}_{\boldsymbol{it}}$ |
| **Frail* x VBC** | $\boldsymbol{Y}_{\boldsymbol{it}}\boldsymbol{=}\boldsymbol{B}_{\boldsymbol{0}}\boldsymbol{+}\boldsymbol{B}_{\boldsymbol{1}}\boldsymbol{\cdot}\boldsymbol{Frail}_{\boldsymbol{it}}\boldsymbol{\cdot}\boldsymbol{VBC}_{\boldsymbol{it}}\boldsymbol{+}\boldsymbol{B}_{\boldsymbol{2}}\boldsymbol{\cdot}\boldsymbol{Age}_{\boldsymbol{it}}\boldsymbol{+}\boldsymbol{B}_{\boldsymbol{3}}\boldsymbol{\cdot}\boldsymbol{Gender}_{\boldsymbol{i}}\boldsymbol{+}\boldsymbol{B}_{\boldsymbol{4}}\boldsymbol{\cdot}\boldsymbol{Race}_{\boldsymbol{i}}\boldsymbol{+}\boldsymbol{B}_{\boldsymbol{5}}\boldsymbol{\cdot}\boldsymbol{Rural}_{\boldsymbol{it}}\boldsymbol{+}\boldsymbol{B}_{\boldsymbol{6}}\boldsymbol{\cdot}\boldsymbol{Disability}_{\boldsymbol{i}}\boldsymbol{+}\boldsymbol{B}_{\boldsymbol{7}}\boldsymbol{\cdot}\boldsymbol{LIS/DE}_{\boldsymbol{it}}\boldsymbol{+}\boldsymbol{B}_{\boldsymbol{8}}\boldsymbol{\cdot}\boldsymbol{Month}_{\boldsymbol{t}}\boldsymbol{+}\boldsymbol{B}_{\boldsymbol{1}}\boldsymbol{\cdot}\boldsymbol{State}_{\boldsymbol{it}}$ |

**Note: Cohort differed due to continuous enrollment requirement for frailty cohort.*
